# Three novel mutations of *STK11* gene in Chinese patients with Peutz–Jeghers syndrome

**DOI:** 10.1186/s12881-016-0339-6

**Published:** 2016-11-08

**Authors:** Hu Tan, Libin Mei, Yanru Huang, Pu Yang, Haoxian Li, Ying Peng, Chen Chen, Xianda Wei, Qian Pan, Desheng Liang, Lingqian Wu

**Affiliations:** 1 The State Key Laboratory of Medical Genetics, Central South University, 110 Xiangya Road, Changsha, Hunan 410078 China; 2 Department of Pediatrics, Xiangya Hospital, Central South University, Changsha, Hunan 410078 China

**Keywords:** Peutz–Jeghers syndrome (PJS), Serine-threonine kinase 11 (*STK11*), Truncating mutation, Severe complication

## Abstract

**Background:**

Peutz–Jeghers syndrome (PJS) is a rare autosomal dominant inherited disorder characterized by gastrointestinal (GI) hamartomatous polyps, mucocutaneous hyperpigmentation, and an increased risk of cancer. Mutations in the serine–threonine kinase 11 gene (*SKT11*) are the major cause of PJS.

**Case presentation:**

Blood samples were collected from six PJS families including eight patients. Mutation screening of *STK11* gene was performed in these six families by Sanger sequencing and multiplex ligation-dependent probe amplification (MLPA) assay. Three novel mutations (c.721G > C, c.645_726del82, and del(exon2–5)) and three recurrent mutations (c.752G > A, c.545 T > C and del(exon1)) in *STK11* were detected in six Chinese PJS families. Genotype-phenotype correlations suggested that truncating mutations trend to result in severe complications.

**Conclusion:**

These findings broaden the mutation spectrum of the *STK11* gene and would help clinicians and genetic counselors provide better clinical surveillance for PJS patients, especially for ones carrying truncating mutation.

**Electronic supplementary material:**

The online version of this article (doi:10.1186/s12881-016-0339-6) contains supplementary material, which is available to authorized users.

## Background

Peutz–Jeghers syndrome (PJS) is a rare autosomal dominant inherited disorder characterized by gastrointestinal (GI) hamartomatous polyps, mucocutaneous hyperpigmentation of the lips, buccal mucosa, and digits. PJS polyps often lead to severe complications, such as intussusceptions and intestinal obstruction. Beyond these symptoms, PJS patients also have an increased risk of cancer at multiple sites, including the GI tract, breast, ovary, testis, and lung [1]. The cumulative lifetime risk is 20, 43, 71, and 89 % at ages 40, 50, 60 and 65 years, respectively [2].

Germline mutations in the serine–threonine kinase 11 (*STK11*) gene on chromosome 19p13.3 were identified as a cause of PJS in 1998 [3, 4]. The gene, 23 kb in size, consists of nine coding exon and one non-coding exon and encodes a 433-amino acid protein, which consists of three domains: the N-terminal non-catalytic domain, the catalytic kinase domain, and the C-terminal non-catalytic regulatory domain. This protein which also acts as a tumor suppressor factor is involved in cell growth, cell polarity, and energy metabolism and plays a pleiotropic role in tumorigenesis [5]. Recent studies showed that mutations in *STK11* can be found in 57–88 % of PJS cases, including point mutations and large genomic deletions/insertions/duplications [6–9].

In this study, we report three novel mutations and three recurrent mutations in *STK11*, which were detected in six Chinese PJS families by Sanger sequencing and the multiplex ligation-dependent probe amplification (MLPA) assay.

## Materials and methods

### Patients

Eight PJS patients from six unrelated Chinese families were enrolled at the Genetics Clinic of the State Key Laboratory of Medical Genetics (SKLMG). Clinical diagnosis for PJS was based on no less than two of these three criteria: characteristic mucocutaneous pigmentation, hamartomatous polyps, and a family history. 100 healthy Chinese individuals (age range from 17 to 40 years, average 22 years) were recruited as controls. Informed consent for this investigation was obtained from all participating PJS patients and parents, and the principles outlined in the Declaration of Helsinki were followed.

### Mutation screening and MLPA assay

Genomic DNA was extracted from peripheral blood by the phenol–chloroform method. The entire coding region and intron–exon boundaries of *STK11* (RefSeq NM_000455) were analyzed by direct Sanger sequencing. Functional signification of novel mutations was predicted by two online software programs, PolyPhen-2 software (http://genetics.bwh.harvard.edu/pph2) and Swiss-Model software (http://swissmodel.expasy.org).

The MLPA test kit (SALSA P101-B1 *STK11*; MRC-Holland, Amsterdam, The Netherlands) was used to detect large genomic deletion/duplication of *STK11* in samples with negative Sanger sequencing results. All operations were conducted using an ABI 3100 genetic analyzer (Applied Biosystems, Carlsbad, CA, USA) following the manufacturer’s recommendations. This experiment was done in triplicates.

## Case presentation

**Fig. 1:**
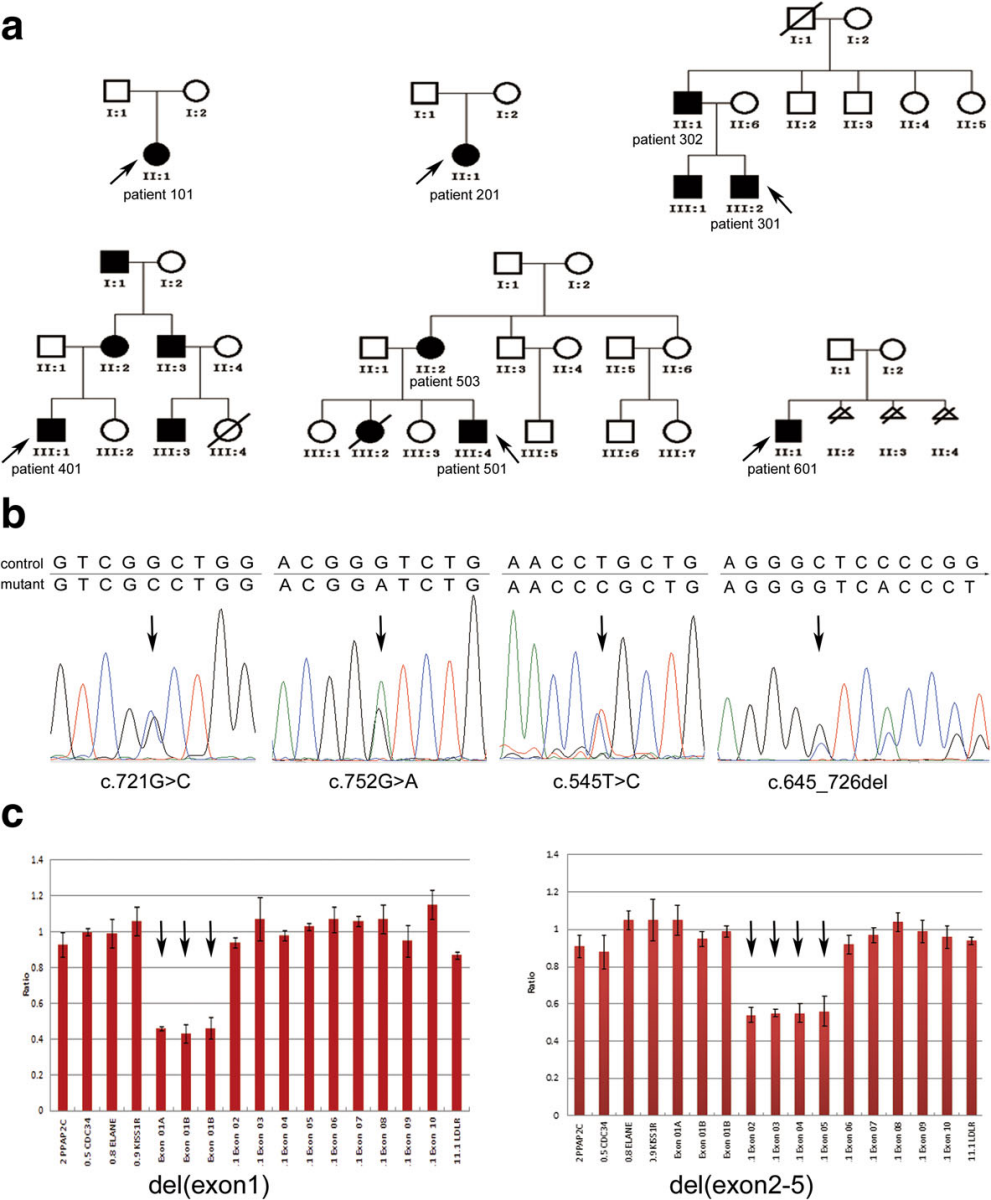
**a** Pedigrees of family 3, 4 and 5 with PJS showed a autosomal dominant pattern and family 1, 2, and 6 were sporadic. **b** Sanger sequencing showed four heterozygous mutations, **c**.721G > C, c.752G > A, c.545 T > C, and c.645_726del. **c** MLPA assay showed two heterozygous gross deletions, del(exon1) and del(exon2-5)

A total of eight patients (2 females and 6 males) from six unrelated families were involved in this study (Table 1). Three of these families (family 3, 4, and 5) showed autosomal dominant pattern and the remaining families (family 1, 2, and 6) are sporadic (Fig. 1a). The patients’ age at *STK11* gene testing ranged from 7 to 38 years old. Five probands in these families underwent the first loparotomy or polypectomy at an earlier age (from 7 to 22 years old), due to abdominal pain or hemafecia. Three out of these patients suffered from some severe complications, intussusceptions and intestinal obstruction. None of these patients enrolled in this study had developed cancer up to the test age except the patient 101 with colonic adenoma. This may be due to a young age (7–45 years old). Detail clinical information is showed in Table 1.

**Table 1:** *STK11* gene mutations and Clinical characteristics of patients with Peutz-Jeghers syndrome

Family	Patient	Exon	Nucleotide change	Gender	Age at test (yr)	Family history	MP	Polyps		FPA (yr)	Intussus -ception	Intestinal obstruction	Cancer
								Localization	Pathology				
1	101	1	Del(exon1)	M	24	No	Yes	colon	hamartomas	10	Yes	No	adenoma
2	201	5	c.721G > C^a^	F	16	No	Yes	NA	NA	NA	NA	NA	NA
3	301	2-5	Del(exon2-5)^a^	M	13	Yes	Yes	Small bowel, colon	hamartomas	7	No	Yes	No
	302	2-5	Del(exon2-5)^a^	M	38	Yes	Yes	NA	NA	NA	No	No	No
4	401	6	c.752G > A	M	30	Yes	Yes	Gastric, colon	hamartomas	22	No	No	No
5	501	4	c.545 T > C	M	7	Yes	Yes	Gastric, colon	hamartomas	7	No	No	No
	503	4	c.545 T > C	F	45	Yes	Yes	NA	NA	NA	No	No	No
6	601	5	c.645_726del^a^	M	9	No	Yes	gastric	hamartomas	8	Yes	No	No

*F* female, *M* male, *NA* no available, *MP* mucocutaneous pigmentation, *FPA* the first polypectomy age

^a^novel mutations

### Results

**Fig. 2:**
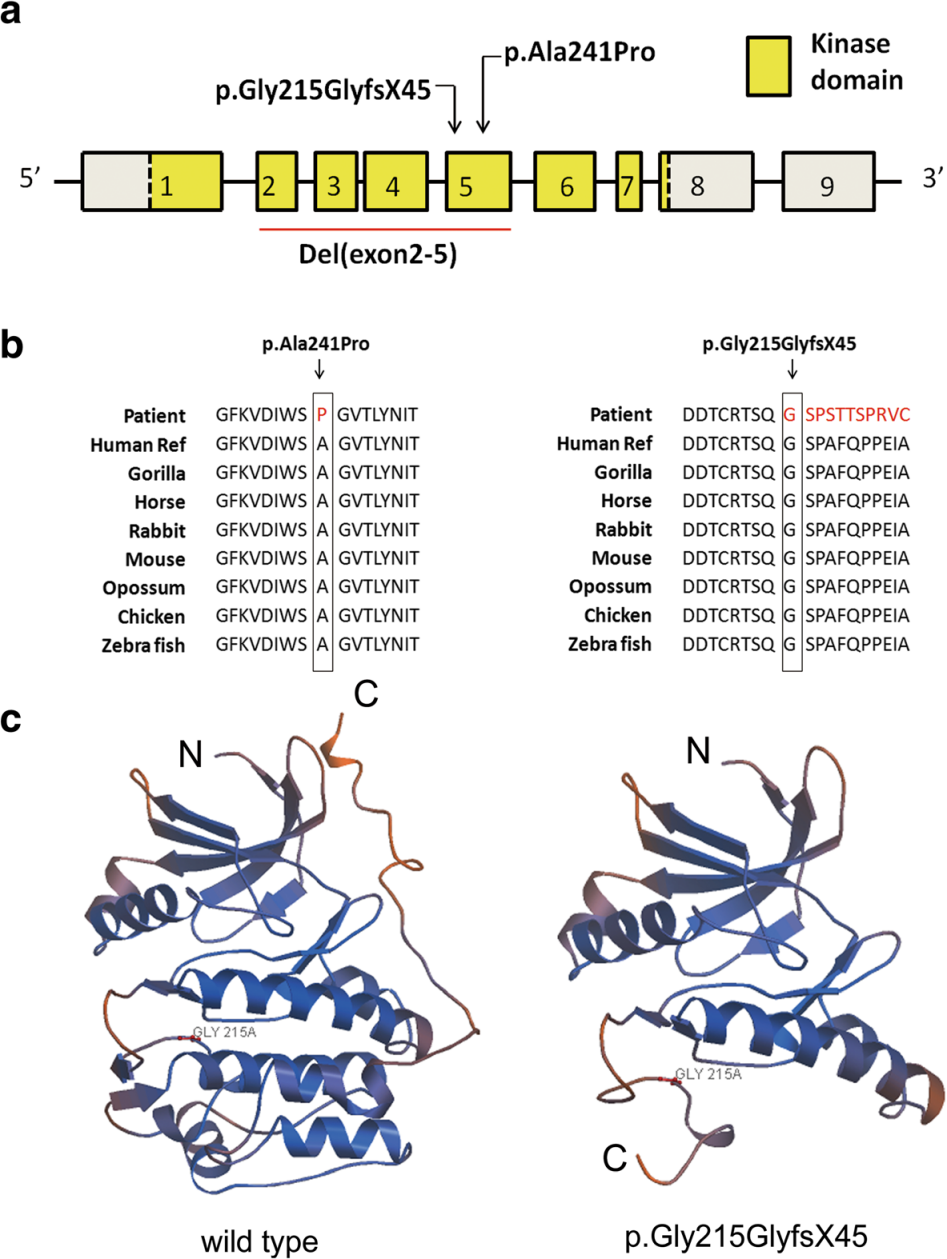
**a** The structure of *STK11* gene. It contains a large kinase domain which spans from exon1 to exon 8. **b** Evolutionary conservation of amino acid residues altered by c.721G > C (p.Ala241Pro) and c.645_726del (p.Gly215GlyfsX45) across different species. **c** The mutant protein (p.Gly215GlyfsX45) was predicted to result in partial loss of the kinase domain and complete loss of the C-terminal domain of the a-helix by Swiss-Model online software compared to the wild type. The *blue Gly215* indicates the mutant site

Direct sequencing of *STK11* gene revealed two recurrent mutation (c.525 T > C and c.752G > A) and two novel mutations (c.721G > C, c.645_726del) in four of six families (Table 1; Fig. 1b). Both of these two novel mutations occurred within the highly conserved kinase domain of *STK11* (Fig. 2a, b). Mutation c.645_726del is *de novo* in sporadic family 6 and c.752G > A and c.545 T > C co-segregated with PJS in multiplex family 4 and 5, respectively. The unaffected parents in sporadic family 2 with c.721G > C were not be tested as DNA samples were not available. The novel missense mutations (c.721G > C) results in the substitution of amino acid in codon 241 (p.Ala241Pro) and was predicted to be “probably damaging” by PolyPhen-2 (Additional file 1: Figure S1). The novel 82-base deletion mutation (c.645_726del) in exon 5 of the *STK11* gene would cause a frameshift change at codon 215, which would introduce a putative stop at codon 259 (p.Gly215GlyfsX45) with partial loss of the kinase domain and complete loss of the C-terminal of the a-helix (Fig. 2c).

Using MLPA analysis, two gross deletions del (exon1) and del(2–5) in the *STK11* gene were detected in the remaining two families (Fig. 1c). Deletion of exon 1 detected in patient 101 has been previously reported and is *de novo* in sporadic family 1. The gross deletion of exon 2–5 found in patient 301 and patient 302 was not found in unaffected members in multiplex family 3, which, as well as novel mutations detected by direct sequencing (c.721G > C and c.645_726del), has not been previously reported in the Human Gene Mutation Database (HGMD), Leiden Open Variation Database (LOVD), NCBI-dbSNP, or Exome Variant Server (EVS). All six variants were not detected in the 100 unrelated normal controls.

## Discussion

Currently, 396 mutations in the *STK11* gene have been detected in patients with PJS or other disorders (HGMD Professional 2016.2), including missense/nonsense mutations (29.8 %), small deletions/insertions/indels (38.9 %), gross deletions/insertions/duplications (20.5 %), splice-site mutations (9.8 %), and complex rearrangements (1.0 %). Many mutations would influence kinase activity of the STK11 protein by preventing it from binding with MO25 and STRAD [10]. STK11 (+/−) mice in previous studies were found to develop gastrointestinal hamartomatous polyposis, suggesting that haploinsufficiency of STK11 is a mechanism of PJS [11].

In the present study, among the six mutations detected in six families with PJS, del(exon1) in family 1, c.752G > A in family 4, and c.545 T > C in family 5 have been previously reported [7, 12, 13]. In family 2, novel missense mutation c.721G > C was detected. Its mutant residue p.Ala241Pro is close to previously identified PJS-causing mutations c.724G > T (p.Gly242Trp) and c.751G > A (p.Gly251Ser), affecting highly conserved residues [12, 14]. The residue altered by p.Ala241Pro are located in the kinase core domain, in which mutations would lead to a loss of the kinase activity and disrupt the function of the STK11 protein, indicating that p.Ala241Pro is most likely disease-causing [15]. In addition, a prediction from PolyPhen-2 online software strongly suggested that it is a pathogenic mutation. Patient 601 in family 6 with typical PJS phenotype carried a novel frameshift mutation c.645_726del (p.Gly215GlyfsX45), which was predicted to generate a prematurely terminated protein with a partial loss of the kinase domain and a complete absence of the C–terminal. Beyond a loss of kinase activity, the truncated protein p.Gly215GlyfsX45 would impair STK11 polarizing activity and the STK11-mediated activation of the AMPK pathway [16]. Moreover, an absence of autophosphorylation and phosphorylation sites Thr336 and Ser428 would disrupt the cell growth suppressive capacity of STK11 protein [5]. In family 3, a novel gross deletion of exon 2–5 were identified in patient 301 and 302. This deletion would be pathogenic as deletions of exon 2–3 and exon 4–5 have been reported to cause a skip of partial exons within the kinase domain [3, 17], which would destroy the structure of STK11 protein and affect its stability by preventing the binding of Hsp90 and Cdc37 [18]. The mutations c.645_726del, del(exon1) and del(exon2–5), as well as previous indentified PJS-causing deletions [7], further demonstrates that haploinsufficiency of STK11 is a mechanism of PJS [11].

In our study, the probands in these six families exhibited the characteristic phenotypes of PJS, among which patient 101, 301, and 601 had complications intussusceptions and intestinal obstruction. As we know, the relationship between the types and sites of variants in *STK11* and the phenotypes of PJS cases has been investigated in several studies. On one hand, individuals with missense mutations in *STK11* typically have a later onset for PJS symptoms [19], but have cancer risks similar to the ones with truncating mutation [20]. In our study, the patients carrying truncating mutations (patient 101, 301, and 601) underwent the first polypectomy of GI polyps and related complications at earlier age (average age = 8.3, standard deviation = 1.2) than the ones with missense mutations (patient 401 and patient 501, average age = 14.5, standard deviation = 7.5). Moreover, we found that more individuals with truncating mutations underwent severe complications than ones with missense mutations, indicating that truncating mutations of *STK11* may have a greater trend to result in severe complications in PJS patients. On the other hand, mutations in exon 3 and exon 6 have been associated with a higher cancer risk [20, 21]. In our study, since no cancer was found in most of these patients, a clear genotype-phenotype correction could not be established. However, colon adenoma detected in patient 101 once again verified a previous reported pathway of hamartoma-(adenoma)-carcinoma in PJS patients [22].

Timely surveillance and early treatment for PJS patients are crucial to improve their quality of life. Current surveillance protocols have two main purposes. One is to detect sizeable GI polyps which could cause severe complications and the other is to detect cancer at an early stage [23]. The potential relationship between truncating mutations of *STK11* and severe complications discovered in this study may help PJS patients improve the quality of life by monitoring the development of GI polyps at an earlier age or at shorter intervals.

## Conclusions

In summary, three novel mutations and three recurrent mutations in *STK11* were identified in Chinese families with PJS, which further broaden the mutation spectrum of *STK11*. This potential relationship between truncating mutations of *STK11* and a higher risk of severe complications would help clinicians and genetic counselors to provide better clinical services for PJS patients.

## Additional file

Additional file 1: Figure S1. Polyphen-2 reports for the pathogenicity of the mutant amino acid residues in STK11. The amino acid substitution p.Ala241Pro was valued “PROBABLY DAMAGING” with the score 0.999 (HumDiv) and 0.971 (HumVar). (TIF 95 kb)

## Abbreviations

Cdc37: Cell division cycle 37
EVS: Exome variant server
GI: Gastrointestinal
HGMD: Human gene mutation database
Hsp90: Heat-shock protein 90
LOVD: Leiden open variation database
MLPA: Multiplex ligation-dependent probe amplification
MO25 (also known as CAB39): The scaffolding protein calcium binding protein 39
PJS: Peutz-Jeghers syndrome
STK11: Serine-threonine kinase 11
STRAD (also known as STRADA): STE20-related kinase adaptor alpha
