# Supplementary figures and images for: Three novel mutations of STK11 gene in Chinese patients with Peutz–Jeghers syndrome
Source: BMC Med Genet. 2016 Nov 8;17:77. doi: 10.1186/s12881-016-0339-6 (PMC5100203; doi:10.1186/s12881-016-0339-6)

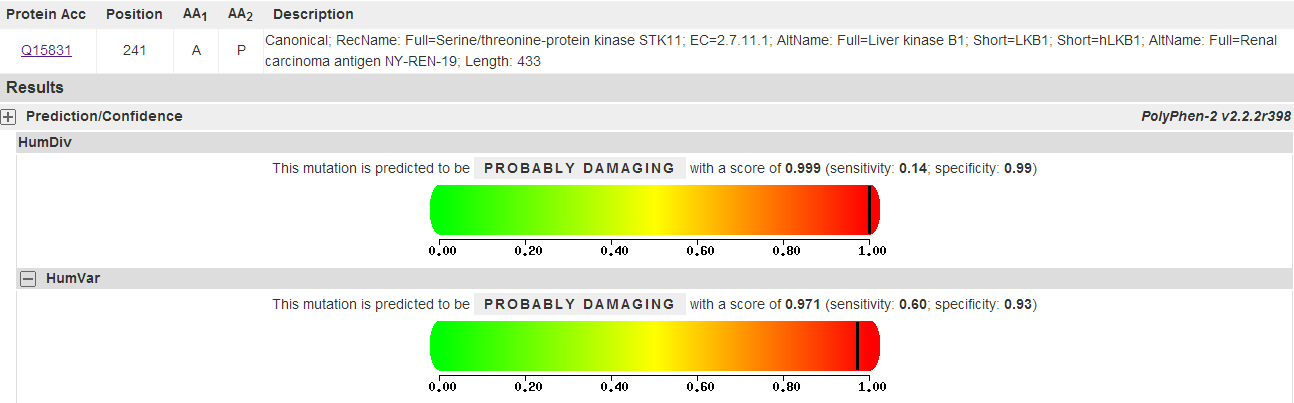

Supplement: Additional file 1: Figure S1. — Polyphen-2 reports for the pathogenicity of the mutant amino acid residues in STK11. The amino acid substitution p.Ala241Pro was valued “PROBABLY DAMAGING” with the score 0.999 (HumDiv) and 0.971 (HumVar). (TIF 95 kb) [file 12881_2016_339_MOESM1_ESM.tif]
